# Low-Cost Online Monitoring System for the Etching Process in Fiber Optic Sensors by Computer Vision

**DOI:** 10.3390/s23135951

**Published:** 2023-06-27

**Authors:** Wenceslao Eduardo Rodríguez-Rodríguez, Jesús Abraham Puente-Sujo, Adolfo Josué Rodríguez-Rodríguez, Ignacio R. Matias, David Tomás Vargas-Requena, Luis Antonio García-Garza

**Affiliations:** 1 Reynosa Rodhe Multidisciplinary Academic Unit, Department of Computational Sciences and Technologies, Computational Systems Academy, Autonomous University of Tamaulipas (UAT), Reynosa-San Fernando Highway, Reynosa 88779, Tamaulipas, Mexico; wrodriguez@uat.edu.mx (W.E.R.-R.);; 2 Department of Electrical, Electronic and Communications Engineering, Institute of Smart Cities (ISC), Public University of Navarre (UPNA), Campus de Arrosadia, 31006 Pamplona, Spain

**Keywords:** fiber optic sensors, SMS, etching, LabVIEW, computer vision, glucose

## Abstract

The present research exposes a novel methodology to manufacture fiber optic sensors following the etching process by Hydrofluoric Acid deposition through a real-time monitoring diameter measurement by computer vision. This is based on virtual instrumentation developed with the National Instruments^®^ technology and a conventional digital microscope. Here, the system has been tested proving its feasibility by the SMS structure diameter reduction from its original diameter of 125 μ until approximately 42.5 μm. The results obtained have allowed us to demonstrate a stable state behavior of the developed system during the etching process through diameter measurement at three different structure sections. Therefore, this proposal will contribute to the etched fiber optic sensor development that requires reaching an enhanced sensitivity. Finally, to demonstrate the previously mentioned SMS without chemical corrosion, and the etched manufactured SMS, both have been applied as glucose concentration sensors.

## 1. Introduction

In the last decades, fiber optic sensor (FOS) development has been growing by research centers, both private and public, owing to their capabilities including small dimensions, multiplexing capability, performance at hazardous conditions and long distances, and immunity to electromagnetic interferences [1]. Therefore, they are preferred in front of other technologies. Due to their optical and physical properties, they have been applied to measure parameters such as refractive index, temperature, strain, pressure, and stress [2,3,4]. Moreover, their implementation for monitoring chemical and biological variables by the deposition of sensitive coatings to a specific analyte is possible [5,6,7]. In FOS, the electromagnetic field that travels through the waveguide in a specific fiber section interacts with the interest surrounding medium, allowing spectral response modulation as the monitoring mechanism. This energy portion is known as the Evanescent Wave (EW) [8]. Its study attracts great interest because it depends on the fiber sensor sensitivity. This means that the longer the EW distance of the fiber that interacts with the interest medium, the higher the sensitivity will be, i.e., the stronger the interaction with the study analyte [9]. The strategy to follow involves modifying the fiber geometry through the sensing section diameter reduction. This can be allowed by the following techniques: heat pulling or etching [10].

The heat-pulling method consists in generating a huge amount of heat either by a flame, electric arc, or a power laser applied to the fiber. At the same moment, both waveguide tails are fixed on translation stages; these axially pull the structure, thus reducing a section and lead to diminishing the core and cladding dimensions at the same proportion, and the developed fiber geometry consists in a taper waist [10,11]. A main drawback to consider in this method is that the fiber tends to break during the process [12]. The etching method comprises a process realized by a chemical reaction via a corrosive agent based on a liquid or vapor, focused an ion beam or dry reactive ion. Liquid etching (also called wet etching) provides a low-cost, feasible, and effective strategy in comparison to heat pulling and the other etchant methods due to the high cost of the experimental equipment needed. Wet etching occurs by fiber immersion into a corrosive solution, which is typically Hydrofluoric Acid (HF). This work focuses its interest on wet etching, simply referred to as etching in this paper.

The state-of-the-art provides knowledge-focused strategies on the etching process in distinct FOS, to reach different sensing applications. Inside the contributions published in this area, there are certain variables to consider, and there are laboratory recommendations for carrying out the experiments, such as room and acid temperature, relative humidity, HF concentration (which leads to a diameter rate variation), fixing the fiber to maintain the structure straight to avoid any bending, and utilizing a container to keep the acid.

The first strategy exposed involves diminishing the diameter sensing region via fiber immersion in HF during a certain duration. S.K Al-Hayali et al. realized etching experimentation with SMS (single mode–multi mode–single mode) sensors for temperature monitoring (coated with a sensitive thin layer fabricated by Copper Oxide/Polyvinyl Alcohol). The structure was diminished until 18 μm using a U-groove to contain the HF at a 40% concentration, obtaining a rate of ~2.84 μm/min [13]. Following the SMS structures, two other contributions have been made. W. E. Rodríguez-Rodríguez et al. reported pH sensor development (coated with a sensitive thin layer constituted by Poly Allylamine Hydrochloride/Poly Acrylic Acid) applying an etch bath based on HF at 40%, keeping the fiber straight using a U-holder manufacturing three different structures with distinct diameters: 64.15 μm, 41.07 μm, and 23.73 μm. They reported that their technique to measure the fiber diameters after the corrosive task was via a computational algorithm based on image triangulation [14]. A. J. Rodríguez-Rodríguez et al. exposed an SMS sensor applied to the gasohol quality control; the structure was fixed onto a channel engraved in a Delrin plate manufacturing the diameter through a buffered corrosive solution, which is a mixture of ammonium fluoride and HF (6:1 volume ratio). The etch task was realized over a 130 min period of reducing the fiber diameter to ~90 μm [15]. M. A. Riza et al. applied an etch bath to FBG (Fiber Bragg Gratings) structures, this being mandatory to its performance as a humidity sensor. Note that these sensors are not sensitive to the surrounding mediums due to the light transmission being confined to the fiber core. So, partial or total remotion of the cladding is necessary. The mentioned contribution reports the utilization of an ABS (Acrylonitrile Butadiene Styrene) holder (manufactured by 3D printing) to straighten the fiber, and an acrylic case to contain the HF at 48%, reaching a diameter of ~10 μm, and etching rate of ~1.69 μm/min [16]. P. Zaca-Morán et al. realized tests in SMF (single-mode fibers) as a sucrose concentration sensor using HF at 48~51%, diminishing the structure until reaching 7.3 μm by a rate of 3.27 μm/min [17]. Following from the previous sensor structure, P. S. Sharma et al. proposed a glucose concentration sensor requiring HF at 40%, containing the acid with a PVC dish. The main contribution of this research has been the demonstration of the temperature, and magnetic stirring effects during the etching, proving that, at higher temperature and stirring speed, the corrosion rate increases [18]. The main drawback of the previous contributions is the absence of some monitoring technique, technology, or parameter that ensures the current measurement diameter during and after the corrosion. To address this issue, a state-of-the-art method presents a second strategy based on online spectral response monitoring which allows to predict the structure diameter. These are mentioned in the next paragraph.

H. J. Kbashi et al. presented an etching process based on two stages: the first one using HF at 30% to remove the cladding, and the second one by HF at 24% to reduce the core diameter, obtaining rates of 1.284 μm/min and 0.184 μm/min, respectively. The experiments were carried out at room temperature [9]. S. Azad et al. showed etching experimentation with MMF (multi-mode fiber) structures applied to humidity sense (coated with a sensitive thin layer fabricated by Zinc Oxide) using HF diluted at a 25% concentration to remove the cladding, obtaining a rate of 0.75 μm/min, and HF at 40% to reduce the core diameter until 28 μm reaching a rate of 1.115 μm/min [19]. In both of the previous contributions, the acid was contained by a Teflon dish and the optical power output monitoring as a fiber diameter measurement mechanism for use during the corrosive task was realized. Following certain fiber structure fabrication characteristics (physical, optical, and geometrical), it is possible to tune the spectral response via the real-time monitoring through the diameter chemical corrosion process. Del Villar et al. demonstrated in LPFG (Long-Period Fiber Gratings) sensors that it is possible to adjust a certain LP (Linearly Polarized) mode appreciated in the spectral response as a dual attenuation band during the etching process, following the Dispersion Turning Point phenomenon, through a specific grating period. The experimentation exposed presents an implementation as a pH sensor (coated with a sensitive thin layer constituted by Poly Allylamine Hydrochloride/Poly Acrylic Acid), allowing to attain a fiber diameter adjustment of up to ~25 μm [20]. Additionally, W.E. Rodríguez-Rodríguez et al. proved that is possible to adjust the SMS sensor diameter through the fourth self-image band presence during the online etch monitoring, defining the sensitive section length according to certain parameters established by the Multi-Modal Interference phenomenon [21]. The experimentation reported an application as an automotive antifreeze sensor, permitting to fabricate three sensors with different diameters. In both of these previous contributions, a U-holder was employed to maintain the fiber as straight along with a PLA (Polylactic Acid) cuvette (fabricated by 3D printing) to maintain the acid.

It is important to mention that in some of the previous contributions the researchers have corroborated the fiber diameter measures through conventional digital microscopes [13,14,16,18,20,21]. This represents a low cost way to measure the fiber diameter. However, other exposed contributions have realized the diameter measurement through SEM (Scanning Electron Microscopy) [9,17,19]. Unfortunately, this last technology represents an economic drawback due its high cost [22]. Therefore, to obtain a specific diameter adjustment in the FOS is fundamental in the implementation of a measurement system in real time during the etching process, to track the process of supervising for which no failure occurs such as evaporation acid, movements or bending in the fibers. A vision measurement system would contribute to monitoring, and avoid the mentioned issues, allowing the researchers its resolution; moreover, it would permit to measure the fiber diameters during the corrosive bath until reaching the desired adjustment.

Therefore, in this contribution, a newfound and low-cost system to adjust the diameter by etching monitoring through a virtual instrument capable of measuring the cross-section during this chemical process via computer vision is proposed. The proposed system has been developed by hardware and software such as a conventional digital microscope and NI^®^ (National Instruments) LabVIEW^®^ (using the NI Vision Acquisition^®^ and NI Vision Development^®^ modules). The work is structured as follows. Section 2 explains the theoretical background of SMS sensors. Section 3 presents the materials and methods. Section 4 describes the methodology followed to design the online monitoring etching process in fiber optic sensors by computer vision in LabVIEW^®^. Section 5 shows a system demonstration when chemical corrosion in an SMS device reaching a diameter of approximately 42.5 μmhas occurred. Moreover, to prove the sensitivity enhancement, another SMS device was fabricated with its original diameter (125 μm); realizing a comparative performance when both are applied as glucose concentration and refractive index sensors. The glucose samples were prepared corresponding to established clinical values to determine in a person’s blood: normal values for non-diabetic, pre-diabetic, and diabetic. Finally, the main conclusions are discussed in Section 6.

## 2. Theoretical Background of SMS Sensors

**Figure 1:**
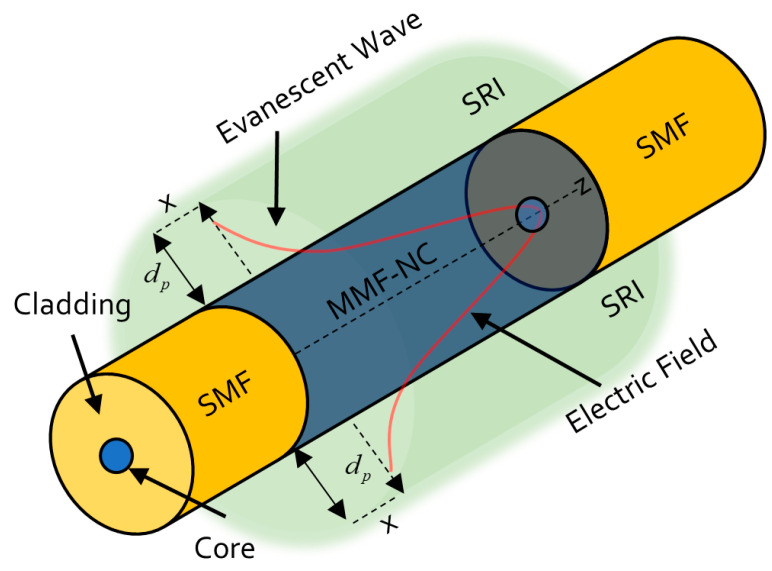
Evanescent wave phenomenon in SMS sensors.

SMS structures are constituted by splicing a multi-mode-no-core fiber (MMF-NC, also known as coreless)-defined segment to two single-mode fiber pigtails. In this kind of fiber sensor, the light that travels through the SMF core is coupled to several propagation modes in the no-core fiber, and thus, a light interaction with the surrounding medium occurs. The previous phenomenon leads to the modulation of its spectral response. The MMF-NC modes have different effective refractive indexes; so, the phase of these modes is distinct when the light is coupled to the core mode of the output SMF segment; causing a constructive and destructive interference as a wavelength function. At the coreless region, it is possible to find light input self-images at specific distances [23,24]. This phenomenon is known as Multi-Modal Interference [25]. The equation that describes the MMF-NC segment (*L*) where these images appear is defined by the following expression.

(1) $$L=p*\left[\frac{n_{nc}D^{2}}{\lambda }\right]$$

where p is the order of the self-image, $n_{nc}$ is the effective refractive index, D is the MMF-NC diameter, and λ is the vacuum’s wavelength. Most of the literature has focused on the fourth-order self-image, that has proved that it possesses a narrow band-pass filter spectral response. Like other fiber sensor configurations, the SMS accomplishes its operation due to the light absorption carried by the EW in the MMF-NC region, allowing the interaction with the surrounding refractive index (SRI), depicted in Figure 1.

The EW is the optical wave that decays exponentially in the perpendicular direction to the MMF-NC/SRI interface. For sensing applications, it is relevant to consider the penetration dept $D_{p}$ of the evanescent wave; here, the distance from the mentioned interface where the electric field amplitude decreases by 1e, defined by

(2) $$D_{p}=\frac{\lambda }{2\pi n_{NC}\sqrt{\sin^{2}\theta_{i}-{\left(\frac{n_{SRI}}{n_{NC}}\right)}^{2}}}$$

where $\theta_{i}\;$is the incident angle in the MMF-NC/SRI interface, and $n_{SRI}$ is the surrounding refractive index. So, a relationship between $D_{p}$ and SRI exists. Therefore, the previous deduction allows to deduce that to enhance the SMS sensor sensitivity is necessary to increase the $D_{p}$ evanescent wave intensity. This can be reached by the MMF-NC diameter diminution. The HF application as a corrosive agent makes the mentioned reduction possible. Once this happens, the etching in the fiber when it is lower in diameter is in the sensitive region, and when higher, it will be the interaction between the interfered high-order modes and the SRI, leading to a higher sensitivity in SMS devices [26]. In addition to the previous SMS sensitivity enhancement background theory, there are two other aspects to consider. The first one is, as the longer the fourth self-image wavelength is established, the higher the sensor sensitivity will be. The second one is that when the SMS sensors operate at an SRI near to the $n_{NC}$, it will reach the higher sensitivity, considering the fact that the SRI cannot be higher than the $n_{NC}$ in the sensors’ implementation. In the case of the present research, $n_{NC}=1$.4525, and an operation wavelength at 800 nm is considered.

## 3. Materials and Methods

**Figure 2:**
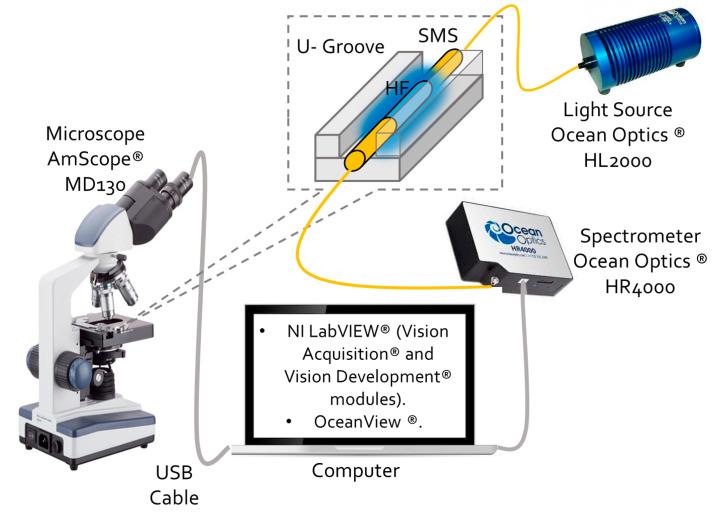
Experimental setup applied for the optical spectrum during the etching and sensor characterization processes.

The online monitoring system for the etching process in fiber optic sensors by computer vision is constituted by the following experimental equipment, software, and fibers. To realize the fiber vision acquisition, the AmScope^®^ MD130 digital microscope was used. The computer vision system was developed by NI LabVIEW^®^ as Virtual Instrument software (VI). Moreover, two NI modules were necessary: NI Vision Acquisition^®^ and NI Vision Development^®^; to acquire the microscope image, and the fiber diameter online measurement during the etching, respectively. The SMS structures used consisted of MMF-coreless FG125LA, spliced to two SMF-S630HP pigtails (core and cladding diameter of 3.5 μm and 125 μm, respectively; NA = 0.12 and operating wavelength 630–860 nm). The fibers were provided by Thorlabs^®^. To demonstrate the sensitivity enhancement due, the diameter reduction by chemical corrosion fabricated two SMS devices with different coreless section lengths. The first one consisted of ~140 mm, as the defined length, to appreciate the fourth self-image at the original fiber diameter (125 μm). The second device has a 7 mm coreless section length; this dimension has been defined to probe the diameter adjustment by computer vision to ~42.5 μm for the function of the fourth-order self-image apparition during the corrosion at ~750 nm optical spectral region. In addition, a HL-2000^®^ white light source and an USB4000 VIR-NIR^®^ spectrometer, both from Ocean Optics^®^, were used. The experimental setup is shown in Figure 2. To differentiate both structures from here, the first device will be named simply as SMS, and the etched structure as eSMS. The eSMS sensitive section was mounted over an acrylic U-Groove to contain the acid. The HF at 48% concentration was provided by Sigma-Aldrich^®^. The experimental results were realized at room temperature. The microscope was isolated with plastic paper to avoid its interaction with liquids.

## 4. Virtual Instrumentation Design for the Online Monitoring Fiber Diameter Measurement during Etching

### 4.1. Image Acquisition Virtual Instrument

**Figure 3:**
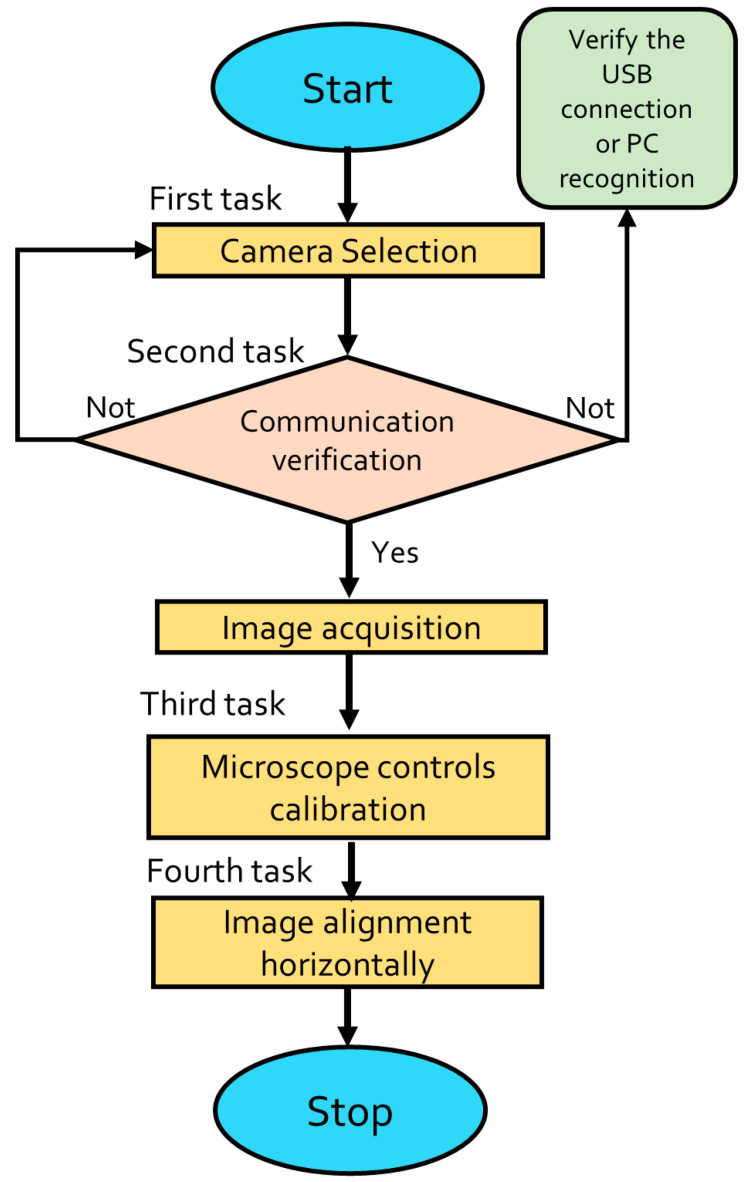
Flowchart describing microscope image acquisition.

**Figure 4:**
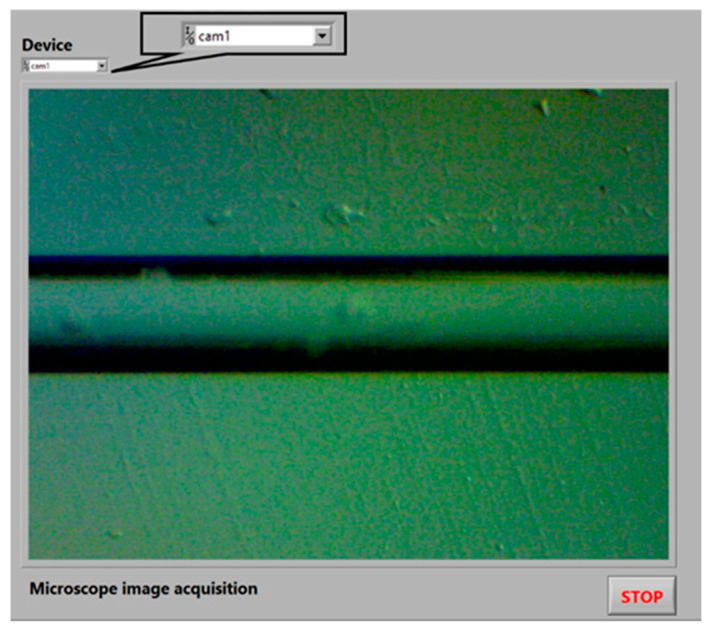
Microscope calibration and image acquisition VI.

This section shows the NI LabVIEW^®^ Virtual Instrument designed for the image acquisition process which is realized through the NI Vision Acquisition^®^ module to obtain the image of the MMF-NC section mounted in the digital microscope. It is detailed next and depicted by the flowchart in Figure 3. The VI is shown in Figure 4.

The microscope image acquisition starts (Start button) running the software. The first task to be carried out by the Device Control is Camera Selection, which is achieved through the IMAQdx Open Camera.vi function. It is mandatory to select a specific device because by default the previous function recognizes all the cameras that have been installed in the PC. Therefore, in this case, the microscope camera is named *cam1.* Once the camera is chosen, the second task is to verify its communication with the PC; this is possible through the image visualization in the VI. The verification of the USB camera connection or the device recognition by the Device Control does not automatically occur. Later, the third task to realize when the fiber image is obtained via the IMAQdx Configure Grab.vi function is the microscope calibration through the mechanical controls, such as brightness adjuster, coarse, and fine focus. Additionally, the task involves selecting the objective lens that permits the image to be clearly identified (in the present case the selected objective lens is the 4 × 0.10). The fourth task is to align the microscope camera horizontally via its manual adjustment, this is demonstrated by a fiber capture visualized in Figure 4. Finally, when all the previous tasks have been realized, the Stop button leads to finishing the image acquisition through the IMAQdx Close Camera.vi function.

### 4.2. NI Vision Assistant^®^ Module Configuration

**Figure 5:**
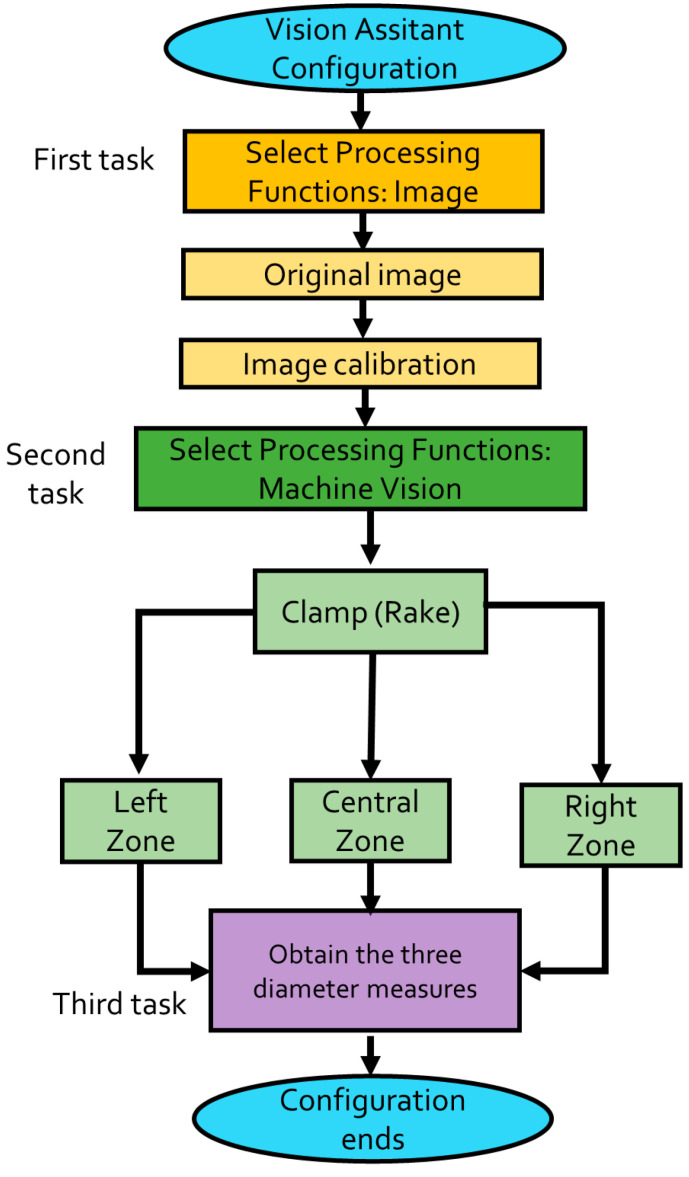
Flowchart describing the NI Vision Assistant^®^ configuration.

**Figure 6:**
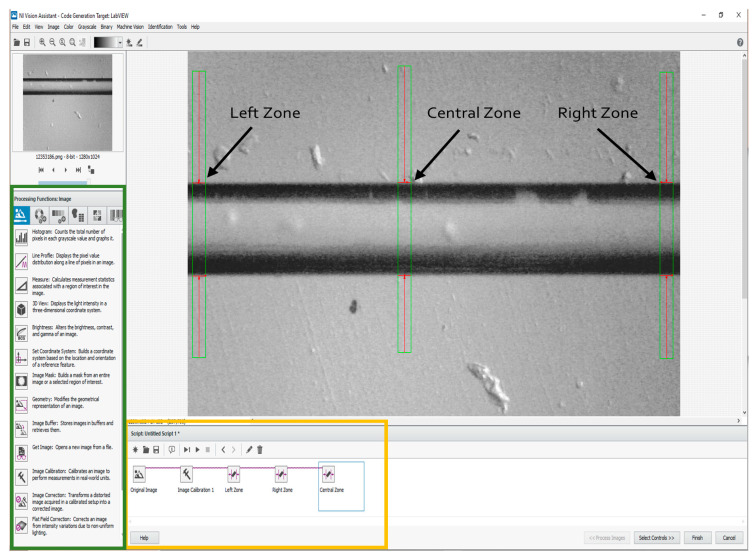
NI Vision Assistant^®^ configuration to the online diameter measurement in three fiber zones: left, central, and right.

Now, it is necessary to first explain the VI developed for the etching monitoring to describe the NI Vision Assistant^®^ module configuration methodology, which allows the fiber diameter to be measured in real time during the process. The description is detailed next, and depicted by the flowchart in Figure 5. The module configuration is shown in Figure 6. The first task to realize is to code the Processing Functions, beginning with the image function. Figure 6 depicts the localized functions and their tools (marked by a green framework). The required tools were selected to be coded in the order at the Script section (marked by a yellow framework, shown in Figure 6).

The Original Image tool permits to visualize the fiber image, one that possesses its original diameter (125 μm) as the reference. The fiber image can be obtained either by a previously saved one or by capturing one by this module. Next, the Image Calibration tool is selected, through a dialog box that permits the selection of the calibration type. For this purpose, the Point Distance Calibration, it directly converts pixel coordinates to real-world coordinates based on a known distance, i.e., the original fiber diameter. Following the dialog box configuration, the stage called Specify Real-World Distances appears, placing two points at the inferior and superior fiber image converting the created distance from pixels to a defined length and units by the user (125, and μm, respectively). The second task is realized by the Processing Function: Machine Vision using its Clamp (Rake) tool permitting the measurement of the distance separating fiber edges. For the desired purpose, this is the mechanism which allows the diameter measurement to be realized, monitoring the distance between the superior and inferior fiber borders. We implemented this tool three times to measure three fiber sections: left, central, and right (denoted by arrows in Figure 6). The reason why we used three measurement zones is in case of failure by processing data or, other factors that interfere with the vision task, (for example bubbles apparition by the HF, or vibration), we ensure the monitoring of the online fiber diameter. The third and last task is to obtain the measurements of the three zones corresponding to the fiber diameter placed in the NI Vision Assistant^®^ module. The previous task allows for the etching supervision through the second VI developed described next.

### 4.3. Online Fiber Diameter Monitoring Virtual Instrument

**Figure 7:**
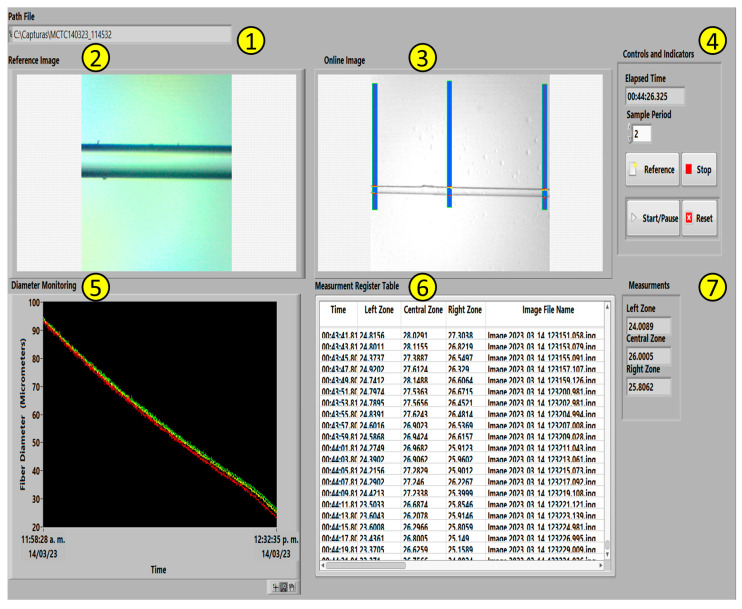
Virtual instrument developed for the online monitoring fiber optics diameter during the etching process.

The third and last task is to obtain the measurements of the three zones corresponding to the fiber diameter placed in the NI Vision Assistant^®^ module. The present VI permits the etching supervision through the second VI developed. It is described next (see Figure 7):

1. The Path File section permits the user to create a new file corresponding to a specific path direction, saving information, such as a reference image, online images obtained during the etchant task, and a.CSV file that contains the information achieved by the Measurement register table (explained later in this section).
2. The Reference Image section shows a reference fiber capture, preferably a fiber image such as the previous etching.
3. The Online Fiber image section shows a real-time image obtained during the etching, denoting the three measurement zones that were established to measure the fiber diameter coded by the NI Vision Assistant^®^.
4. The Controls and Indicators section allows the management and supervision of certain tasks for the user such as the Reference control capturing a fiber image from the microscope as a reference; the Start/Pause and Reset, both controlling a chronometer to monitor the etching duration time visualized by the Elapsed Time indicator. The Sample Period control, as its name suggests, establishes the measured sample period in seconds. The Stop button aborts the VI execution.
5. The Diameter Monitoring section visualizes, via a graphic indicator, the online diameter considering three section measures: left (red), central (yellow), and right (green); indicating in the X-axis, the date and elapsed time; in Y-axis, the fiber diameter (μm).
6. The Measurement Register Table section registers the mandatory information corresponding to the process, such as elapsed time; left, central, and right online fiber diameter measurements; and the image file name. This last one is the respective name (titled by date and experimentation time) assigned to the saved image captured during the etch task stored in the path file created by the VI.
7. The Measurements section shows the measurements obtained by the NI Vision Assistant^®^ specifying the correspondent measure zone (left, central, and right).

## 5. Experimental Results

### 5.1. Developed System Demonstration Adjusting the SMS Diameter by Etching

**Figure 8:**
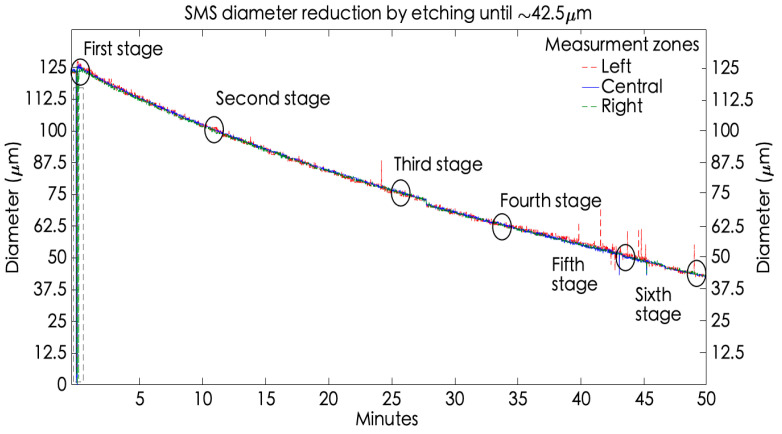
Online monitoring of a single-mode fiber during the etching.

This section shows the developed system performance when it is implemented to monitor the etching process in the MMF-NC section of an SMS sensor with an original diameter of 125 μm until it reaches a diminution of ~42.5 μm. The fiber manufacturing is described by six stages described next. Figure 8 shows the etching monitoring process indicated by circles at each stage. The fiber optic image obtained during the etching and the corresponding information of each stage, such as diameter measured and elapsed time; are presented in Figure 8 and Table 1, respectively.

**Figure 9:**
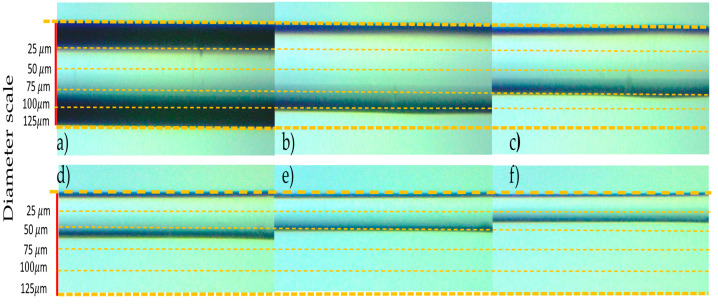
SMS captures obtained by the six etching stages: (**a**) first, (**b**) second, (**c**) third, (**d**) fourth, (**e**) fifth, and (**f**) sixth.

The first stage is constituted by placing the fiber segment over an acrylic U-groove on the microscope, acquiring the image by the VI described in Section 4.1. To begin with the chemical corrosion, it is recommended to capture a fiber reference image, visualized in Figure 9a). This reference image allows measures about its original diameter to be acquired in three zones: left = 123.56 μm, central = 123.56 μm, and right = 123.66 μm. Now, the etching begins with the HF deposition, and as expected, the image has turned out of focus, making it necessary to change the image focus through the coarse adjustment microscope knob. This is denoted in Figure 8 by a gray dashed framework. Later, the three zone measurements were recovered, showing the diameter diminution. After that, at the 10 elapsed minutes, the fiber diameter decreased by about a fifth. This was considered the second stage, showing the measures: left = 101.68 μm, central = 102.99 μm, and right = 101.67 μm (see Figure 9b). Next, the process continued until the fiber reached a diminution of ~75 μm at the 20 elapsed minutes; this is considered the third stage, obtaining the next measurement zones: left = 75.46 μm, central = 76.73 μm, and right = 77.13 μm (observe Figure 9c). Later, considering an elapsed time of 33 min, the fiber decreased by a diameter of close to half, reaching the following measures: left = 62.87 μm, central = 62.8 μm, and right = 62.21 μm (shown in Figure 9d)). This scenario is called the fourth stage. Then, the fifth stage occurs at 43 elapsed minutes, and the fiber diameter measures are left = 50.8 μm, central = 50.52 μm, and left = 50.95 μm (observe Figure 9e). Finally, at the 50 elapsed minutes, the MMF-NC reaches the desired diameter, and the measures obtained from the monitoring system are left = 42.34 μm, central = 42.26 μm, and right = 42.77 μm (see Figure 9f). This is the sixth and last stage.

**Figure 10:**
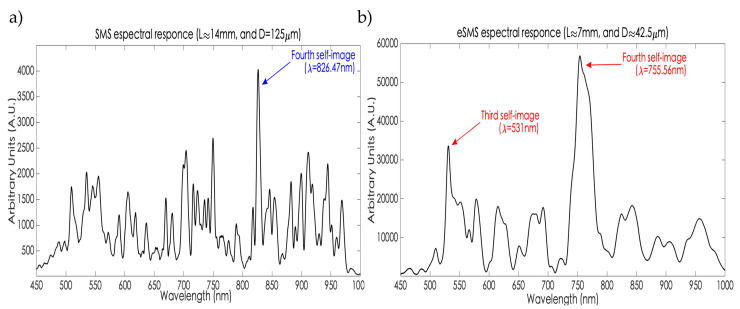
Transmission spectral response of the devices: (**a**) SMS without etching (D = 125 μm), (**b**) etched SMS (D ≈ 42.5 μm).

Until this section, it is relevant to mention the following statement obtained by the experimental trial. The previous experiment corroborates that the etching rate is not constant, it starts diminishing when the etching happens. As is shown in Table 1, the current rate from each stage descends in comparison with the previous one. From the first stage to the second stage, i.e., when the fiber diameter has been dimished by ~25 μm, the current rate is the highest during the experiment at 2.14 μm/min. After that, the rate diminishes. In the last etching stage (the sixth), when the fiber has been diminished by ~82.5 μm, the final obtained rate is 1.65 μm/min. Therefore, in conclusion, the rate is not the same during the complete etching experiment. Considering the fact that the HF deposition only occurred once the test began, this phenomenon is because the acid concentration diminishes during the process by external factors such as the temperature and the relative humidity. Finally, the transmission spectral response of the developed SMS structures is exposed (see Figure 10). In Figure 10a, the SMS transmission response considering physical parameters, such as L≈14 mm and D = 125 μm is visualized, with the fourth self-image being visualized at 826.47 nm.

As was mentioned in Section 2, this self-image order is preferred as the operational reference to be applied in sensing tasks, due to it being the narrower band with the higher intensity. Unfortunately, when the SMS operates at the visible light region, the sensitivity is lower in front of those sensors that realize their performance at the near-infrared region. So, to overcome this issue, the etching allows improvement in the sensitivity due the EW penetration depth which will as high as the diameter will be diminished, i.e., the stronger the interaction will be between the fiber and the surrounding medium. However, it has a cost as is shown in Figure 10b. The eSMS transmission spectral response with L ≈ 7 mm and D ≈ 42.5 μm probes that as long as the diameter be reduced, the wider the self-images will be leading to difficulty in their visibility and implementation as a sensor. This is the reason why an etched SMS has been fabricated to this cross-section measurement. Figure 10b also shows the third and fourth self-images, at 531 nm and 755.56 nm, respectively.

### 5.2. SMS Structures Application as Glucose Concentration Sensor

Once the developed system has demonstrated its performance manufacturing an etched SMS structure with a diameter of ~42.5 μm, this section demonstrated the sensitivity enhancenment achieved compared with an SMS device with its original diameter (125 μm). For this purpose, both structures are applied as glucose concentration sensors. The relevance to monitoring this biological paramater is because it is necessary for the proper functioning of the organs and tissues. Glucose is obtained from food and is absorbed into the bloodstream from the small intestine [27]. The pancreas helps to monitor the blood glucose levels, every time carbohydrates are digested, where certain cells in this organ release insulin in the blood. Then, insulin guides the glucose into fats, liver, and muscle, allowing it to be used as the primary energy source in the human body. Insulin is the hormone that regulates blood sugar levels. Between meals, the blood sugar must return to normal levels. These blood levels are measured by mg/dL units, and are the following:

- Normal blood glucose concentration (Gc) levels (before a meal) are 70 mg/dL to 100 mg/dL, or two hours after the start of a meal are <140 mg/dL.
- Pre-diabetes (before a meal) levels are 100 to 125 mg/dL, or two hours after a meal are 140 mg/dL to 199 mg/dL.
- Non-normal Gc levels that indicate diabetes (before a meal) are >126 mg/dL, or two hours after a meal are >200 mg/dL.
- Dangerous blood Gc levels >240 mg/dL mean hyperglycemia, commonly known as high blood sugar. This condition is only a problem for diabetic individuals because they suffer from dysfunctional insulin.

**Figure 11:**
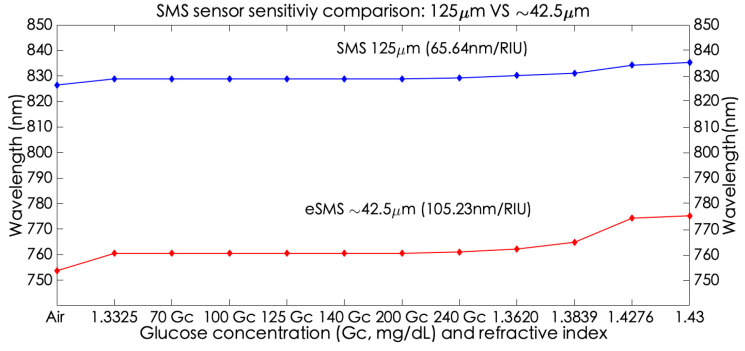
SMS sensor sensitivity comparison: 125 μm vs. ~42.5 μm.

Therefore, to apply the SMS devices as glucose level sensors, glucose solutions in distilled water were applied. In order to characterize the sensors’ performance, another five samples were elaborated on by known refractive indexes considering the wavelength operation at 800 nm: Sample 1 is distilled water (S1), Sample 2 is Ethanol (S2 = 1.3575), Sample 3 is a mixed solution constituted by Ethanol/Ethylene Glycol (60%/40%; S3 ≈ 1.3823, Sample 4 is a mixed solution constituted by Ethanol/Ethylene Glycol (2%/98%; S4 ≈ 1.42), and Sample 5 is Ethylene Glycol (S5 = 1.4263). The obtained results by the characterization of both sensors are presented and visualized in Table 2 and Figure 11. As well as for the other sensor, the same performance from the S1 to the 200 mg/dL Glucose Concentration (Gc) solution, a non-wavelengtht change was obtained. This is because the biochemicals are made with low values of SRI (1.33–134) [28]. Although minimum refractive index changes occur in these samples, these are indetectable by the spectrometer Limit of Detection (LOD) that does not allow to measure these small index variations. Despite this scenario, both sensors reach the point of sensing a small wavelength shift when the 240Gc solution is measured, in front of the previous measurements of 0.37 nm and 3.59 nm, in SMS and eSMS, respectively. Here is where the sensitivity enhancement, small but noticeable, is appreciated. So, the eSMS is capable of detecting hyperglycemia.

Finally, in order to demonstrate the higher sensitivity of the sensors according to Sample 5, which has the higher refractive index, the sensitivity obtained is 65.64 nm/RIU, and 105.23 nm/RIU, to SMS and eSMS devices, respectively. Concluding that, when the SMS diameter is reduced to almost a third part, the sensitivity increases by a factor of 1.60.

## 6. Conclusions

In this work, a novel online monitoring system for the etching process in fiber optic sensors has been presented that realizes the diameter measurement through computer vision based on a conventional digital microscope, and the NI LabVIEW^®^ software considering two NI modules: the Vision Acquisition^®^ and Vision Development^®^. Against other technologies that utilize expensive technology such as Scanning Electron Microscopy; our proposal represents a low-cost alternative due to it being constituted by hardware and software that can be affordable by any private or public research center.

Despite that uncontrollable variables exist during the etching process that impact on the rate, such as temperature and relative humidity leading to diminish the HF concentration, the system has demonstrated a performance feasible in allowing to diminish an SMS structure until the desired diameter is reached: ~42.5 μm. We have defined this adjustment as when the fiber cross-section measure reaches the lower dimensions which might compromise the structure fragility, manipulation, and time life. It is possible to realize the etchant task by increasing the temperature, as well as by stirring the acid to increase the rate; but this would lead to increasing the solution vaporization, making it hazardous to work with [11]. This risk could be avoided by using an HF at lower concentration. Regarding the behavior shown by the online monitoring system we developed, it is remarkable to mention two important aspects. The first one is that the system required an image-focus adjustment by the coarse microscope knob on only one occasion, this solely being when the HF was deposited over the fiber.

Although there is still work to be carried out, our proposal opens the door to the manufacture of fiber optic sensors in laboratories via the National Instruments^®^ technology and its computer vision modules, for example, when the D-shaped etching in fibers is mandatory to maintain its characteristic fiber geometry. In the future, this research group intends to improve the proposed technology, to combine the etching tracking through computer vision with the second strategy mentioned in the state-of-the-art strategy via the spectral response monitoring obtained by the structures, thus allowing to predict and verify the structure diameter. Both strategies would strengthen the fiber sensors’ manufacture via the etching process with the goal of improving its sensitivity to realize chemical and biological sensing applications.

On the other hand, the eSMS sensor reached the capability of detecting hyperglycemia in an appreciable spectral response. In order to improve the sensitivity of the glucose concentration in SMS sensors, it is necessary to follow three strategies. The first one is to increase the diameter diminution to improve the sensitivity; in combination with the second one, realize the thin film deposition based on polymers, oxides, or nanoparticles with a higher refractive index than the MMF-NC. The third strategy consists of immobilizing a recognition element biologically capable not only of improving the sensitivity, but to become a sensor capable of realizing a selectivity of the biological compound (in this case glucose) and obtain the level concentration of different samples. In this case, our early research career (author, and correspondent author) opens the door to a new investigation topic.

## Figures and Tables

**Table 1:** SMS etching process stages.

Stage	Left Zone (μm)	Central Zone (μm)	Right Zone (μm)	Elapsed Time (min)	Current Rate(μm/min)
First	123.56	123.56	123.66	-	-
Second	101.68	102.99	101.67	10	2.14
Third	75.46	76.73	77.13	25	1.89
Fourth	62.87	62.8	62.21	33	1.84
Fifth	50.8	50.52	50.95	43	1.7
Sixth	42.34	42.26	42.77	50	1.65

**Table 2:** Refractive index and wavelength shift of each glucose concentration and test samples.

	Air	S1	70 Gc	100 Gc	125 Gc	140 Gc	200 Gc	240 Gc	S2	S3	S4	S5
**RI**	1	1.3285	-	-	-	-	-	-	1.3575	1.3823	1.42	1.4264
**SMS** **(nm)**	826.5	828.86	828.86	828.86	828.76	828.86	828.86	829.23	830.15	831.26	834.20	835.3
**eSMS** **(nm)**	753.66	761.25	761.25	761.25	761.25	761.25	761.25	764.84	762.19	764.84	774.28	775.2
